# Editorial: Evolutionary Feedbacks Between Population Biology and Genome Architecture

**DOI:** 10.3389/fgene.2018.00329

**Published:** 2018-08-21

**Authors:** Tariq Ezaz, Scott V. Edwards

**Affiliations:** ^1^Institute for Applied Ecology, University of Canberra Canberra, ACT, Australia; ^2^Department of Organismic and Evolutionary Biology and Museum of Comparative Zoology, Harvard University Cambridge, MA, United States

**Keywords:** microRNA, nearly neutral theory, vertebrate, CNVs, transposable element (TE), natural selection

Joe Felsenstein famously quipped in 1988 that “systematists and evolutionary geneticists don't often talk to each other,” laying bare the schism between macroevolutionary thinking, such as building phylogenies, and population genetics (Felsenstein, 1988). Thirty years ago, in its early phase, studies of genome evolution were already beginning to incorporate population genetics when comparing the DNA of distantly related species (Dover and Flavell, 1982), but the field was far from mature. Today this schism between population genetics and genome evolution is much healed, being bridged by novel statistical methods for detecting natural selection (Kreitman and Akashi, 1995), as well as to the monumental book by Michael Lynch (2007), “The Origins of Genome Architecture,” which in turn built upon the foundational nearly neutral theory of Tomoko Ohta and Motoo Kimura (Ohta, 1973, 1992). This Frontiers Research Topic celebrates this increasing infusion of population biology perspectives into studies of genome evolution, which has facilitated key advances in our understanding of how eukaryotic and microbial genomes evolve and the evolutionary forces influencing their structure.

This research topic includes 10 papers that encompass a wide range of model and non-model systems from bacteria, plants, crustaceans, and vertebrates. In addition to linking population biology and genome architecture, these chapters also study diverse components of the genome, including organelle and nuclear genomes, karyotypes, sex chromosomes, RNA transcripts, small non-coding RNAs, and repetitive elements, to understand genome evolution, speciation, and population divergence. For example, Nagai et al. combined mitochondrial and candidate sex determining gene Sox3 sequences to demonstrate speciation events as well as gene flow among 16 populations of frogs from Japan. In a similar approach that delved into interactions between the mitochondrial and nuclear genomes in two avian and one crustacean species, Sunnucks et al. demonstrated how genomic architecture might facilitate better understanding of co-adoption of mitonuclear interactions and enhance biochemical efficiencies of oxidative phosphorylation. Potter et al. present another approach by combining genome sequencing data with cytogenetics to understand chromosome rearrangements leading to speciation and population divergence. Using a unique Australian native marsupial, the rock wallaby, Potter and colleagues demonstrate the value of combined approaches of cytogenetics and genomics to understand evolution of genome architecture as driver of speciation. Addressing the variability of LINE element composition in vertebrates, Ruggiero et al. resequenced 13 genomes of green anole (*Anolis carolinensis*) from two populations and found high variation in the frequencies of polymorphic LINE elements, concluding that large effective population size and negative selection together curb the proliferation of LINEs in this species. In another inquiry into repeated sequences, Samelak-Czajka et al. expand on a clever and accurate approach for quantifying CNVs in *Arabidopsis* genomes, offering a promising approach to quantifying these widespread structures in plants. Each of these studies illustrates how molecular and population processes can interact in unexpected ways to shape the structure of eukaryotic genomes, an interdisciplinarity that requires consideration of the population context in which molecular variation is being studied.

Other studies in the Research Topic attempt to bridge population biology and genome evolution at macroevolutionary scales. Hua and Bromham tackle the perennial question of whether rates of lineage diversification are linked to rates of genome evolution and discuss a wide range of results and theoretical connections between the two. França et al. bring a genomic microscope to examining lineage diversification by reviewing the role of microRNAs in altering gene expression between species, thereby playing a potential role in phenotypic evolution and disease. An Miao An et al. turn attention to lineage fusion when studying phenotypic and molecular introgression in oaks in China. They find both morphological and molecular signals of introgression in this complex, in some cases leading to genetic swamping and likely mediated by habitat degradation. Bobay and Ochman update our knowledge of broad trends in bacterial genome architecture, showing how genome size is a product of the interaction of nearly neutral forces of drift and mutation bias toward deletions and how the interplay of selection and drift cause deviations from a strict correlation between genome size and gene number. Finally, Romiguier and Roux synthesize information on the interactions among of GC-biased gene conversion, recombination and natural selection in modulating GC-content in eukaryotes. They illustrate the interplay between micro- and macroevolution by showing how variation in GC-content among lineages can strongly bias our estimation of phylogenies, natural selection, and the extent of codon bias.

The articles in this Research Topic offer a useful snapshot of how research in genome evolution naturally incorporates insights and theories from population genetics, but also some of the challenges of doing so. To what extent should macroevolutionary models remain phenomenological, or instead incorporate the minutiae of evolutionary forces to predict observed patterns today (Hua and Bromham)? How can modern genomics effectively update the classical theories of chromosomal speciation (Potter et al.), or abundance of transposable elements (Ruggiero et al.), and apply them to natural populations? Today, coalescent methods of phylogenetic inference (Liu et al., 2015; Xu and Yang, 2016) have largely answered Felsenstein's quip. Similarly, the nearly neutral theory is widely applied on a genome-wide scale today (e.g., Yi, 2006; Akashi et al., 2012; Gossmann et al., 2012; Denisov et al., 2014), but even more noteworthy is the wide range of species and settings in which it is applied (e.g., Figuet et al., 2016; Chen et al., 2018). As illustrated by the articles in this Research Topic, population biology thinking has been shown to be useful in most areas of genome evolution where researchers care to apply it, from the evolution of sex chromosomes, microRNAs or transposable elements, to GC-content and genome size. This Research Topic shows the breadth of situations, both genomic and ecological, in which population-thinking is helping us to interpret genome evolution. And, in doing so, few can say today that genome evolutionists and population biologists rarely talk to each other.

## Author contributions

All authors listed have made a substantial, direct and intellectual contribution to the work, and approved it for publication.

## Conflict of interest statement

The authors declare that the research was conducted in the absence of any commercial or financial relationships that could be construed as a potential conflict of interest.
